# Vacuum-Assisted Breast Biopsy System: No Innovation Without Evaluation

**DOI:** 10.7759/cureus.12649

**Published:** 2021-01-12

**Authors:** Sherif Monib, Soumitra Mukerji, Sonia Narula

**Affiliations:** 1 Breast Surgery, West Hertfordshire Hospitals NHS Trust, St Albans, GBR; 2 Radiology, West Hertfordshire Hospitals NHS Trust, St Albans, GBR

**Keywords:** breast cancer- minimally invasive diagnosis- vacuum-assisted biopsy-vacuum assisted excision- stereotactic vacuum - calcifications

## Abstract

Background

Vacuum-assisted breast biopsy (VABB) has recently been gaining more popularity as a modality to reach the final diagnosis, especially in indeterminate breast lesions, resulting in a decreased number of surgical interventions and unnecessary follow-ups.

Objective

While our primary aim was to look into the outcomes of the VABB technique, our secondary aim was to assess the impact of the method on changes in patients’ management.

Patients and methods

This study was a retrospective database analysis of vacuum-assisted biopsies (VABs) carried out at our breast unit during the period between January 2011 and January 2018. All our cases were image-guided; the caliber of vacuum-assisted needles used was 8 gauge (G) and 11 G. Patient demographics, lesion characteristics, and outcomes were retrieved from patients’ notes and the hospital database.

Results

A total of 122 female patients were included in the analysis, out of whom 41.8% (51 patients) were screen-detected, and 58.1% (71 patients) were symptomatic presentations. The mean lesion size on imaging was 14.8 mm (SD: 12.6); 50% (61 patients) had stereotactic vacuum-assisted breast biopsy (SVAB), and 50% (61 patients) had ultrasound-guided vacuum-assisted breast biopsy (US-VAB). Post-procedure histology was upgraded in 19.6% (24 patients), downgraded in 18.8% (23 patients), and remained unchanged in 61.4% (75 patients).

Conclusion

VABB is a safe and efficient procedure for the diagnosis and management of indeterminate and suspicious breast lesions. It provides an adequate amount of tissue, which can help in upgrading or downgrading histopathologically diagnosed patients, thereby decreasing the need for surgery.

## Introduction

In 1989, Parker started performing percutaneous biopsies, using a core needle and an automatic deployment device. In 1995, Mark Retchard, a medical device engineer, and Fred Burbank, a radiologist, developed the technique of vacuum-assisted breast biopsy (VABB) to overcome the shortcomings of core biopsies and improve the accuracy of biopsies of microcalcification (MCC) in adipose breast tissue by using an automatic biopsy gun [1,2].

Soon, VABB was proven to be an excellent alternative to surgical biopsy, and it came to be associated with high diagnostic accuracy and low patient discomfort [3]. Given that larger tissue samples are obtained by VABB, and the histological diagnosis is more accurate, the role of the vacuum-assisted biopsy (VAB) was extended to therapeutic procedures [4].

Advantages of VABB over standard core biopsy include the fact that it reduces sampling errors, imaging-histological discordance, as well as re-biopsy rates. Indications of VABB include 1) clarification of B3 lesions [atypical ductal hyperplasia (ADH), atypical lobular hyperplasia (ALH), lobular carcinoma in situ (LCIS), papillary lesions, radial scar, flat epithelial atypia]; 2) excision of benign lesions with curative intent; 3) re-biopsy of discordant cases lacking correlation of suspected diagnosis and histology; 4) MCC following B1/B3/B4 result on core biopsy; and 5) diagnostic excision of papillary lesions and radial scars diagnosed on core biopsy.

In this study, we aimed to look into the outcomes of the VABB technique, focusing on safety as well as the impact of the technique on patient management.

## Materials and methods

Study setting

This study was conducted at the St Albans breast unit, West Hertfordshire Hospitals NHS Trust, UK.

Ethical considerations

The Ethical Committee approval was not required as this was a retrospective study.

Study design 

We conducted a retrospective database analysis of symptomatic as well as screen-detected patients who had VABs or excisions in our breast unit during the period from January 2011 till January 2018. All patients had triple assessments including clinical breast examination followed by a digital mammogram and/or breast ultrasound scan, followed by an initial 14 G core biopsy. MRI was carried out for a select group of patients.

The results of triple assessments were discussed among our multidisciplinary team (MDT), and the decision to proceed with VABB was in line with the MDT in all cases. Patient demographics, lesion characteristics, and patient outcomes were retrieved from patients’ notes, the Clinical Record Interactive Search (CRIS) system, and the picture archiving and communication system (PACS). All patients were seen in the clinic one week after the procedure for clinical examination and to discuss histological findings and management plans.

## Results

**Figure 1:**
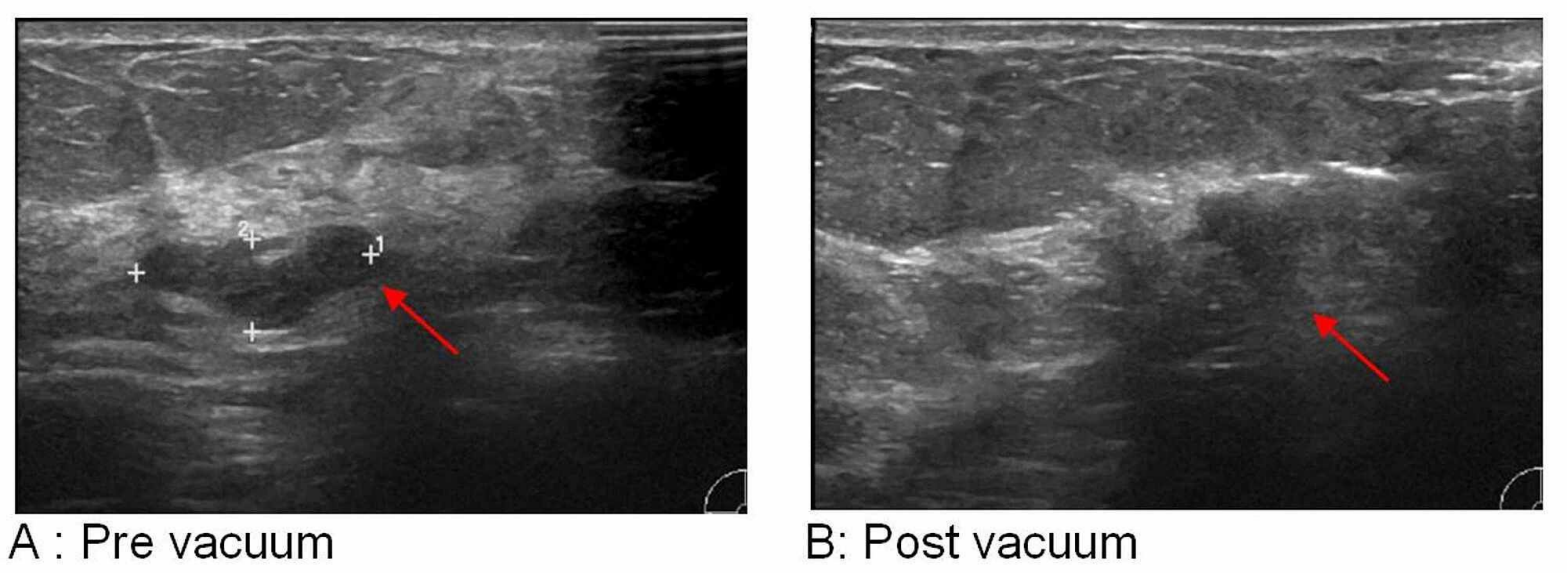
Left breast ultrasound scan of a 9 o’clock-located 16-mm fibroadenoma with LCIS in a 60-year-old female patient LCIS: lobular carcinoma in situ

**Figure 2:**
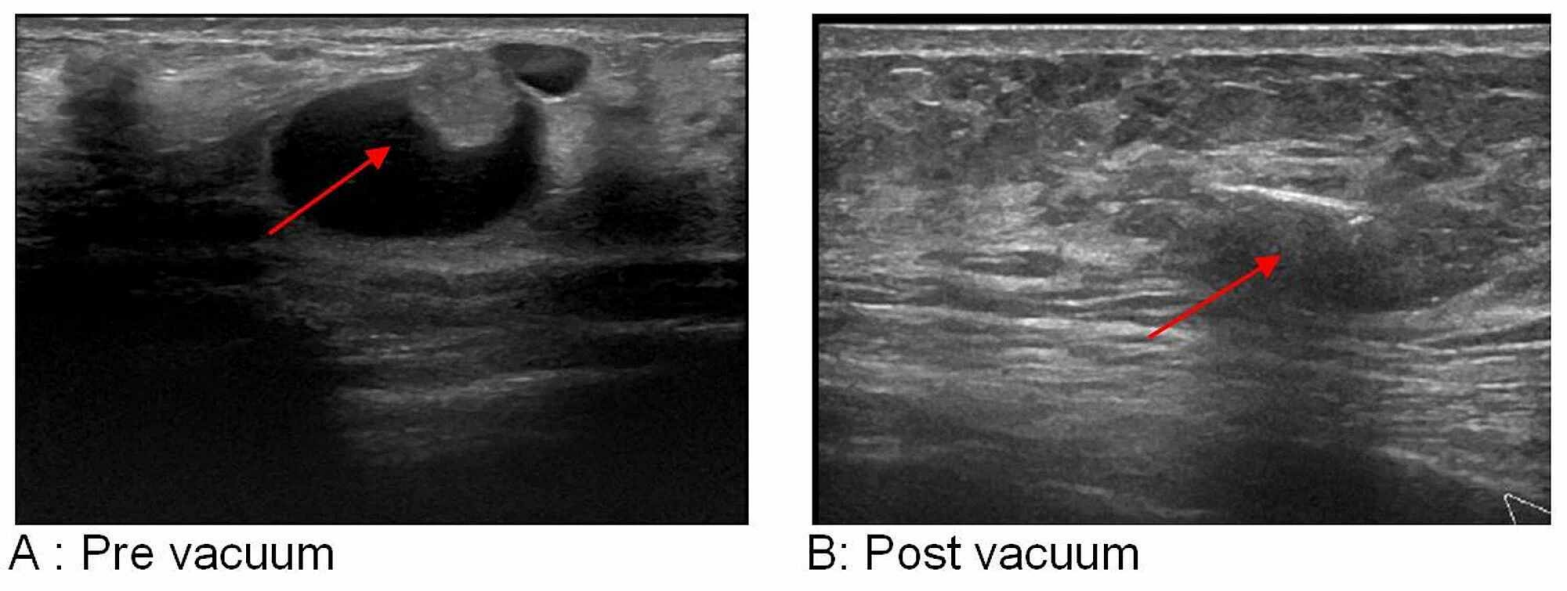
Right breast ultrasound scan of a 10 o’clock-located 18-mm intracystic papilloma in a 44-year-old female patient

**Figure 3:**
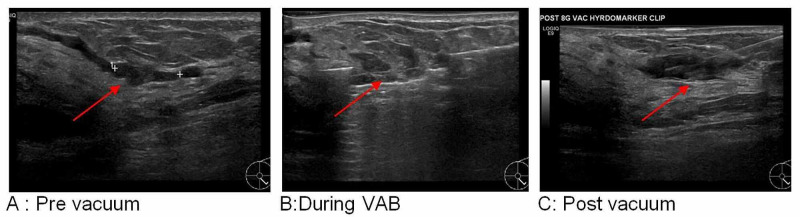
Left breast, 5 o’clock, 13-mm intraduct papilloma in a 43-year-old female patient VAB: vacuum-assisted biopsy

**Figure 4:**
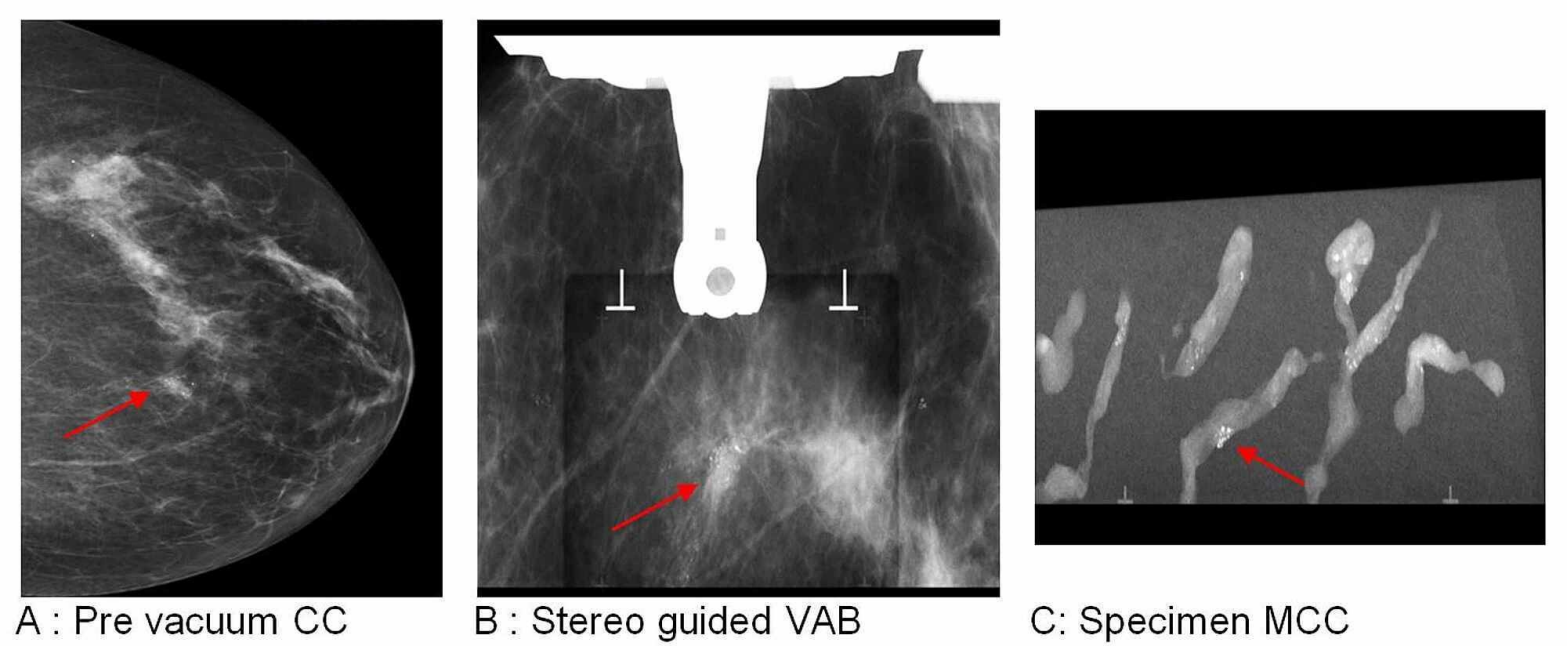
Left breast, 12 o’clock, 34-mm DCIS in a 59-year-old female patient DCIS: ductal carcinoma in situ; CC: craniocaudal; VAB: vacuum-assisted biopsy; MCC: microcalcification

**Figure 5:**
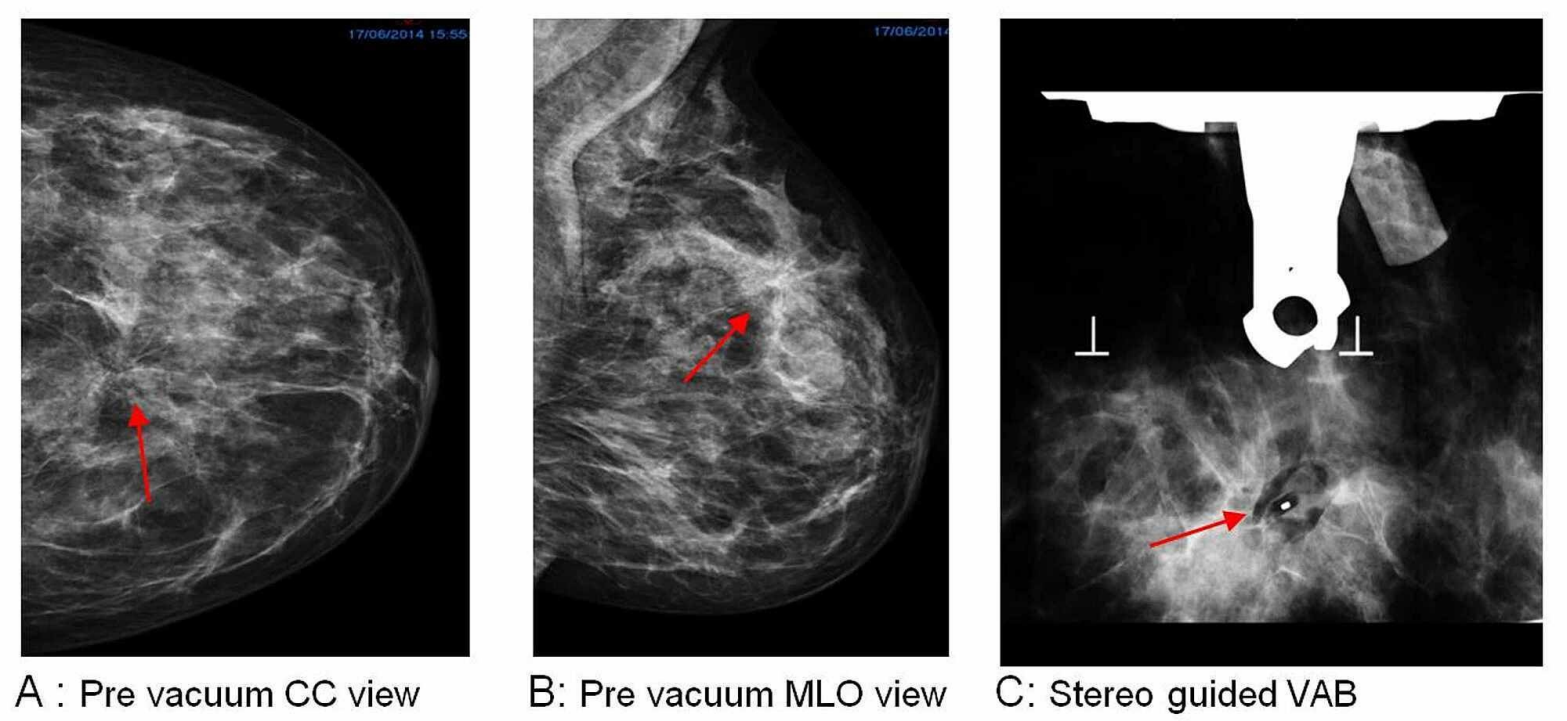
Left breast, 12 o’clock, 25-mm radial scar in a 55-year-old female patient CC: craniocaudal; MLO: mediolateral oblique; VAB: vacuum-assisted biopsy

We included 122 females patients in the analysis. Among them, 41.8% (51 patients) were screen-detected and 58.1% (71 patients) were symptomatic presentations; 52.4% (64 patients) had right-side lesions, while 47.5% (58 patients) had left-side lesions. Mammographic findings revealed that 41.8% (51 patients) had MCC, 9% (11 patients) had mammographic distortion, and 46.7% (57 patients) had mass lesions; the mean lesion size on imaging was 14.8 mm (SD: 12.6) (Figure 1, Figure 2, Figure 3, Figure 4, Figure 5).

All procedures were image-guided; 50% (61 patients) underwent stereotactic vacuum-assisted breast biopsy (SVAB), and 50% (61 patients) had ultrasound-guided vacuum-assisted breast biopsy (US-VAB). Vacuum-assisted excisions (VAEs) using an 8 G needle were carried out for 68% (83 patients), and VABs using an 11 G needle were carried out for 31.9% (39 patients) of the cohort. Our overall immediate complications rate was 3.2%; 2.4% (three patients) developed a hematoma, which was treated conservatively, and 0.8% (one patient) had skin breakdown, which was also treated conservatively.

Histological findings

Pre-procedure histology grading was B1 in 4.9% (six patients), B2 in 6.5% (eight patients), B3 in 81.1% (99 patients), B4 in 0.8% (one patient), B5a in 2.4% (three patients), and B5b in 4% (five patients) (Table 1).

**Table 1:** Pre-procedure histological grading

Histological grading	% (number of patients)
B1	4.9% (6 patients)
B2	6.5% (8 patients)
B3	81.1% (99 patients)
B4	0.8% (1 patient)
B5a, B5b	2.4% (3 patients), 4% (5 patients)

Post-procedure histology was upgraded from B1, B2, B3, and B4 to B5a or B5b in 18% (22 patients); it was downgraded from B3 to B2 in 18.8% (23 patients), and was unchanged in 63.1% (77 patients) (Table 2, Table 3).

**Table 2:** Post-procedure histological grading

Histological grading	% (number of patients)
Upgraded from B1, B2, and B3 to B5a or B5b	18% (22 patients)
Downgraded from B3 to B2	18.8% (23 patients)
Unchanged	63.1% (77 patients)

**Table 3:** The breakdown of upgraded pathology

Pre-vacuum pathology	Post-vacuum pathology
B1: 2.4% (3 patients)	B3: 1.6% (2 patients), B5b: 0.8% (1 patient)
B2: 0.8% (1 patient)	B3: 0.8% (1 patient)
B3: 13.9% (17 patients)	B5a: 9% (11 patients), B5b: 4.9% (6 patients)
B4: 0.8% (1 patient)	B5b: 0.8% (1 patient)

Impact of VAB on management

Of note, 31.1% (38 patients) were discharged; 9% (11 patients) required a follow-up mammogram or US scan with or without re-biopsy, while 24.5% (30 patients) required five-year surveillance mammograms, and 28.6% (35 patients) required definitive surgical treatment in the form of wide local excision or a mastectomy.

## Discussion

Indeterminate (B3) breast lesions represent a heterogeneous group of abnormalities; its incidence is 5% in screen-detected patients, which is slightly higher than that in symptomatic patients [5]. While repeat biopsy does not always provide a definitive diagnosis, VABB not only provides enough tissue for assessment but also can provide definitive treatment at the same time.

There are no strict guidelines regarding the maximum size of lesions that can be safely excised using VABB; however, excisions are usually limited to lesions under 30 mm due to time constraints and reasons related to patient comfort. In our cohort, the mean lesion size was 14.8 mm (SD: 12.6). Park et al. have concluded in their analysis that it is safe to use VABB to resect lesions of <3 cm [6].

VABs are always carried out under image-guidance; selection of the best modality is based on the modality's ability to show the index lesion and where the lesion is easily accessible for the procedure [7]. SVAB was introduced by Burbank and Parker in 1996 as a diagnostic tool to evaluate suspicious lesions visible on mammography [2]. It was found to be accurate as a method of open surgical biopsy with lower complication rates; also, mammographic changes after surgical biopsies, such as architectural distortion, parenchyma scar, calcifications, fat necrosis, and asymmetric glandular tissue defects have been well described and may mimic the mammographic signs of malignancy [8]. US-VAB was first performed by Zannis et al. in 1998 [2]. It is considered a good alternative in cases with mammographically occult breast lesions and for excision of benign lesions, such as fibroadenoma, papilloma, and radial scars [7]. MRI-guided VABB is also an accurate method for diagnosing breast lesions not seen on mammogram or US scan [9]. Recently, tomosynthesis-guided VABB (TVAB) has also been proven to be able to biopsy small architectural distortions and MCC with high accuracy [10]. Of note, 53.6% of our cases were SVABs, while 46.3% were US-VABs.

VABB needles come in 8 G, 11 G, or 14 G diameters; the 14 G needle is the least invasive, and it can collect 40 mg of tissue per insertion. The 11 G needle can collect 100 mg of tissue, and hence it can be used to completely resect lesions of <1 cm. The 8 G needle can collect 250 mg of tissue, and hence it is capable of resecting palpable breast lesions smaller than 3 cm [2]. Other needles used are the 13 G, 9G, and 7G monobloc excision needles [11]. In our cohort, we used 8 G for VAEs and 11 G for VABs. den Dekker et al. found that six 9 G VAB specimens are enough to reach a final histopathological diagnosis in 95% of cases [12]. In our practice, we always aim to take 12 specimens for VAB and between 12-20 specimens for VAE to achieve complete excision.

In our experience, for diagnostic VAEs, adequate sampling can be ensured by undertaking lesion excision in two steps: inner ring for the lesion and outer ring (with clock-face circumferential sampling) for lesion margins, to emulate surgical excision. This gives the pathologist enough tissue for the analysis of the lesion as well as its margins.

Following VAB for MCC/ductal carcinoma in situ (DCIS), around 10% of patients are usually found to have invasive diseases [13]; this upgrade is essential for surgical planning as not only patients with invasive disease but also those with mass-forming DCIS will require a sentinel lymph node biopsy at the time of definitive surgery [14]. In our cohort, post-procedure histology was upgraded from B2, B3, and B4 to B5a or B5b in 18% (22 patients), downgraded from B3 to B2 in 18.8% (23 patients), and was unchanged in 63.1% (77 patients); out of the initial 99 B3 lesions that were downgraded, eight patients were discharged, and six patients had planned surveillance. It is worth mentioning that by using the VABB technique, we diagnosed 19 additional cancers (15.5%), out of which 11 (9%) were non-invasive, and eight (6.5%) were invasive cancers.

Unfortunately, VABB is not without complications; to minimize the incidence of possible complications, VABB is not recommended in cases with scattered MCC, lesions close to the skin, areola and nipple complex, or chest wall due to the difficulty in access and increased risk of skin dehiscence. While Park et al. reported a complication rate of 2.5% including bleeding, hematoma formation, skin injury, and dimple formation, or even pneumothorax [15], Hu et al. reported pain in 22.6%, hematomas formation in 9.7%, and ecchymosis in 3.2% [16]. US-VAB is associated with a slightly higher risk of bleeding when compared to SVAB, which can be attributed to the lack of breast compression during the US-guided procedure [17].

Our cohort’s overall immediate complications rate was 3.2% (4/122 patients), with 2.4% (3/122 patients) developing a hematoma, which was treated conservatively, and 0.8% (1/122 patient) with skin breakdown, which was also treated conservatively. It is worth mentioning that most of the patients who developed complications had mostly superficial lesions. Our low complication rate was due to careful procedure technique, use of a combination of local anesthetic lidocaine with adrenaline (1 in 200,000) to reduce the risk of bleeding, use of excision guidance (ultrasound versus stereotactic) to best suit the lesion position, and avoiding access close to nipple and chest wall. The close proximity to the skin can sometimes be overcome by excising from above than from below, such that the cutting edge of the device is facing the depth of breast tissue away from the skin, thereby reducing the risk of skin dehiscence. Infiltration of local anesthetic and saline can be helpful in increasing the depth between the lesion and the chest wall, thereby enabling excision of posteriorly placed lesions close to the chest wall. We also feel that excising the lesion in the inner (lesion) and outer ring (margin/periphery) enhances the specimen yield and also gives a margin excision equivalent, thus mimicking surgical excision, especially in lesions such as papillomata and radial scar.

Other possible complications include clip migration, which is seen more with superficial lesions, and high specimen number [18]. In our cohort, we did not come across significant clip migration cases. Also, inadequate sampling or missed lesions can significantly affect patient outcomes; therefore, intra-procedural, early post-procedural mammography, as well as specimen radiography are required to ensure adequate sampling [15,19]. As skin injury was also reported as a possible complication, Berná-Serna et al. used a simple, safe technique to prevent skin injury during VABB by inserting a spinal needle between the skin and the mass [20].

Perretta et al. found US-guided VAE to be highly successful with a complete excision rate of 93.61%; they also found that circumscribed margins, regular shape, parallel orientation, and the absence of posterior features were favorable US features associated with complete excision [21]. van de Voort et al. also found VAE to be safe and effective for benign lesions of up to 50 mm; moreover, their patients reported good cosmetic outcomes [22].

While all our patients and most cases in the literature have been females, Atallah NG has reported the first case of US-VAB for a man with breast MCC, which was found to be a case of DCIS [23]. Also, Qu et al. found VAE to be a feasible and minimally invasive approach for the treatment of gynecomastia [24]. VABB is associated with significantly lower procedural costs when compared to open surgery, without compromising on the quality of patient care [25].

Limitations

Our study has some limitations. One of them was the low number of patients, which limited the statistical powering of our study. Another limitation is the fact that we are still in the process of following up on patients.

## Conclusions

VABB is a safe and efficient procedure for the assessment and management of indeterminate and suspicious breast lesions, which not only avoids open surgical procedures in the majority of cases but also helps to avoid histological underestimation, resulting in significant cost savings. It is generally well tolerated by patients and is especially suitable for papillary neoplasms, lobular neoplasia (LN), atypical intraductal epithelial proliferation (AIDEP), flat epithelial atypia, and radial scar excision, with an acceptable complication rate.
